# Fentanyl and Spiradoline Interactions in a Place-Conditioning Black-White Shuttle-Box

**DOI:** 10.3390/ph401101

**Published:** 2010-12-24

**Authors:** Richard H. Rech, Shannon L. Briggs, David J. Mokler

**Affiliations:** 1 Department of Pharmacology/Toxicology, Michigan State University, East Lansing, MI 48824, USA; E-Mail: rech@msu.edu (R.H.R.); 2 Department of Environmental Quality, State of Michigan, Lansing, MI 48909, USA; E-Mail: briggss4@michigan.gov (S.L.B.); 3 Department of Biomedical Sciences, College of Osteopathic Medicine, University of New England, 11 Hills Beach Road, Biddeford, ME 04005, USA

**Keywords:** fentanyl, spiradoline, place preference, place aversion, two-compartment, shuttle-box

## Abstract

Rats were trained for multiple sessions in a place-conditioning shuttle-box to explore motivational interactions of mu and kappa opioid agonists, specifically fentanyl reward and spiradoline aversion. In Phase 1, groups of rats received various doses of mu or kappa agonists, or placebo, testing for preference or aversion. Group **A** always received saline SC before 15-minute sessions. Group **B** received fentanyl SC (0.003, 0.006, 0.012 mg/kg), Group **C** received low and medium doses of agonists SC, and Group **D** received spiradoline (0.3, 0.6, 1.2 mg/kg) SC during Training Sessions 1-4, rats being restricted to the drug-associated compartment. Rats received saline when restricted to the placebo-associate compartment and on test days with access to both shuttle-box compartments. In Phase 2 of the study, Training Session 5, Combinations of mu and kappa agonists were substituted in Groups **B**, **C**, and **D**. Dose-related preference to fentanyl and aversion to spiradoline occurred during Test Sessions 1-4. During Test Session 5, fentanyl preference in Group **B** was suppressed by spiradoline, rats in Group **C** had a saline-like response to combined agonists, and spiradoline aversion in Group **D** was attenuated by fentanyl. These findings suggest that combined doses of mu and kappa agonists, while additive for antinociception, offset the rewarding and punishing effects of each other.

## 1. Introduction

In the last three decades much research has been conducted on behavioral effects of mu-opioid-receptor (MOR) agonists and kappa-opioid-receptor (KOR) agonists. For example, Shippenberg and colleagues reported extensively on antinociceptive and motivational responses of these drug classes [1-6]. Generally, testing of MOR agonists in place-conditioning (PC) paradigms showed conditioned place preference (CPP) that was attenuated by combination with a KOR agonist. PC training with a KOR agonist resulted in a conditioned place aversion CPA). Concerning antinociceptive effects (ANC), both agonists were active when administered singly. With combined agonists, additive ANC was usually observed in somatic pain test models [7,8], whereas synergistic (supra-additive) ANC responses occurred in visceral pain tests on pairing the agonists [9,10,11] (see below for more details).

Our laboratory initiated tests for ANC in domestic cats using visceral stimuli (colorectal distension, CRD), seeking reliable opioid pain relief in this species [12]. Felines react to MOR agonists with a manic-like excitation, compromising effective treatment [13]. We tested butorphanol (a mixed MOR, KOR agonist [14,15]) in cats exposed to CRD, and observed effective ANC. Butorphanol-treated subjects remained calm for the first hour, responding to petting with purring, as opposed to a deliriant-type reaction to the MOR agonist oxymorphone. But, as the ANC effect began to wane, the cats became irritable to touch and noise, resisting being handled [16]. We also combined butorphanol and oxymorphone in cats, and found enhanced ANC with CRD, along with reduced side effects of both agonists, including the secondary irritable response to butorphanol alone.

A class of KOR-agonist analgesics (arylacetamides, including U-50,488, enadoline, spiradoline, and U69593) was described by von Voigtlander and Lewis [17] as highly efficacious and selective analgesics acting at KOR-1 sites. We chose enadoline and spiradoline to be tested with fentanyl (potent and selective MOR agonist [18]) for ANC and PC studies in rats. Fentanyl and spiradoline, given singly, induced maximal efficacy for ANC in cold-water tail-flick (CWTF, somatic pain test model) and CRD. Combined agonists induced mainly additive ANC in CWTF, with reduced side effects of each class [7]. Rats tested with the combination in CRD (visceral pain test model) exhibited a synergistic ANC, with reduction of side effects of each agonist [9].

Pretreatment with selective MOR or KOR antagonists attenuated selectively the CWTF ANC of fentanyl and spiradoline, respectively, as anticipated. But pretreatment of rats with the antagonists against ANC in CRD did not show selective antagonisms. That is, in the visceral pain model, both antagonists decreased the ANC of the MOR and KOR agonists non-selectively.

One of us (RHR) had developed an early version of an unbiased PC procedure that included food appetitive responses to *d*-amphetamine and *d*-fenfluramine [19]. Fasted rats were tested in a 4-alley black-white x-maze. Subjects trained on *d*-amphetamine developed CPP, while those trained on *d*-fenfluramine manifested CPA. Both drugs caused anorexia, rats mouthing but not eating pellets that were left in the alleys, while no pellets were observed after saline injection.

Briggs, as a graduate student, decided to use the x-maze to explore fentanyl and enadoline PC interactions as a part of her MSU dissertation research: *Interactions of mu- and kappa-opioid agonists*, which was successfully defended in 1996. Briggs observed a significant CPP in rats trained on fentanyl in the x-maze, which was attenuated by a low dose of enadoline. However, this enadoline dose alone, trained for a PC response, induced only a non-significant trend for CPA. Our enadoline supply had been exhausted by extensive ANC testing, and Briggs also was faced with a deadline for her thesis defense. Thus, she was not able to extend the x-maze PC studies at that time.

Investigations of MOR- and KOR-agonist PC interactions have usually been prompted by searching for new therapies to treat MOR-agonist addiction (see Discussion). While this topic is of interest to us, it was not our major goal in Dr. Briggs's x-maze study or in this shuttle-box PC work. Rather, our main purpose was to test the hypothesis that the aversive (dysphoric) effect of a KOR agonist could be suppressed by combination with a MOR agonist. This KOR-agonist aversion is the main deterrent to the clinical use of the KOR analgesics such as spiradoline and enadoline in humans (see Discussion). Thus, proof of the above hypothesis may lead to greater patient acceptance of the combined agonists, especially for those suffering chronic visceral pain.

## 2. Experimental Methods and Materials

### 2.1. Subjects and Drugs

Male Sprague-Dawley adult rats, weighing from 220 to 270 grams at the start of the study, were divided into four groups of 6 rats each: **A**, **B**, **C**, and **D**. Table 1 lists the schedule of training and testing of drug and placebo treatments for all groups over a six-week period.

Training and test trials were 15 min in duration. All groups received saline injections SC during the Pre-Training Session to establish baseline placebo responses from the results of the Pre-Training Test.Group A (placebo group) received only saline in all training and test trials.

During Test Sessions 1-4 (Table 1) Group **B** received low-dose fentanyl, medium-dose fentanyl (MDF), and high-dose fentanyl to establish a dose-response pattern of conditioned place preference (CPP). The second high dose of fentanyl was calculated to enhance the final CPP determination. During Training Session 5 the combined high-dose fentanyl and low-dose spiradoline (HDF + LDS) tested the hypothesis that fentanyl CPP may be attenuated by addition of spiradoline.

Group **C** received two dose levels of fentanyl (Training Sessions 1 and 2) and two dose levels of spiradoline (Training Sessions 3 and 4) to compare PC effects with those of Groups **B** and **D**, as well as to establish baseline values for the Group **C** Test Day 5, combined medium-dose fentanyl and medium-dose spiradoline (MDF + MDS). This latter test was calculated to determine the relative strengths of medium doses of the combined agonists as to interaction of opposing motivational effects.

Group **D** was trained to three dose levels of spiradoline (sessions 1-4) for a dose-response CPA pattern, the high dose repeated (HDS2) to maximize the final CPA score. Group **D** rats then received combined high-dose spiradoline and low-dose fentanyl (HDS + LDF, Test 5) to assess the capacity of the low dose of fentanyl to reduce the CPA score of the high dose of spiradoline.

The drug dose levels of fentanyl, 0.003, 0.006, 0.012 mg/kg, and spiradoline, 0.3, 0.6, 1.2 mg/kg, were based upon previously established antinociceptive (ANC) dose-response patterns [7,9]. Fentanyl ANC reached peak activity at 15 min post-injection SC and began to decay at about 30 min post-injection. Spiradoline ANC peaked near 30 min post-injection SC and began to decay about 50 min post-injection. Therefore, fentanyl was injected 15 min before placing a subject into the shuttle-box, and spiradoline was injected 30 min prior to placement.

Fentanyl citrate was purchased from Elkins-Sims, Inc. (Cherry-Hill, NJ, USA). Spiradoline was generously supplied by P. L. von Voigtlander (Upjohn Co., Kalamazoo, MI, USA). The drugs were dissolved in normal saline. The rats and procedures were approved for this study, per NIH standards, by the Animal Use and Care Committee, Michigan State University.

### 2.1. Conditioning Apparatus, Training and Testing Parameters

**Figure 1:**
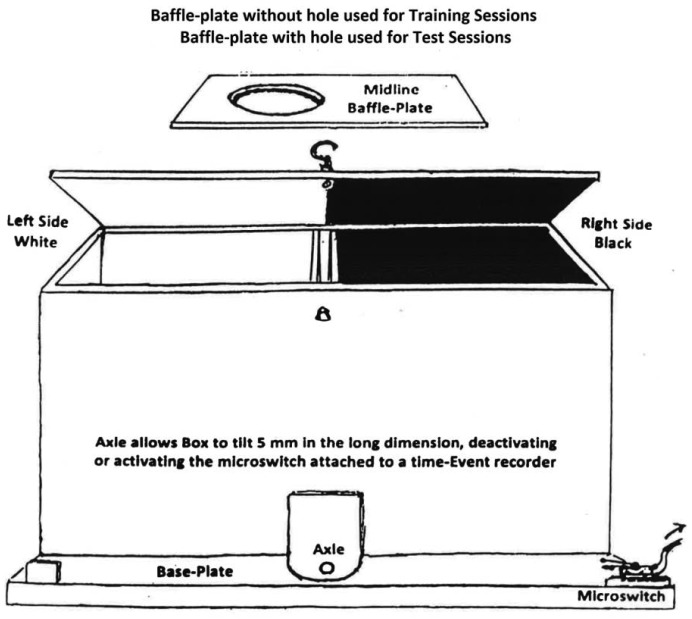
Place-conditioning Shuttle-Box.

Two shuttle-boxes were constructed with two compartments each, 35 cm long by 13 cm wide by 13 cm high. The compartments were joined at the narrow walls on one side, in which 7 cm diameter circles were cut, one cm above the floors. In each box one compartment was painted black with a mesh floor, the other painted white with a smooth floor. A rectangular baffle-plate, black on one side and white on the other, was inserted between connecting walls to restrict an occupant to one side or the other on training days. On test days the baffle-plate was removed, allowing subjects access to both compartments. See Figure 1 for details of the shuttle-box design.

An axle fitted under the connecting walls caused the box to tilt a few mm in the long dimension as a rat moved from one compartment to the other. This action activated or deactivated a micro-switch attached to one of the box. The micro-switch contacts were connected to an electrical time-event recorder that registered times of tilting by a needle displacement running on pressure-sensitive paper. The percent of time of a 15-min trial that a subject spent in each compartment on test days was read from the tapes by the researcher at the end of each trial. Three of the six rats in each group were restricted to the black compartment and the other three to the white compartment on drug-training days. For each subject this compartment was designated the drug-associated compartment (DAC). On placebo-training days subjects were injected with saline and restricted to the opposite (placebo-associated, PAC) compartment. Since Group **A** received only saline in either compartment, for it the term “drug-associated compartment” is a misnomer. However, the term was retained with Group A for the sake of conformity.

### 2.2. Analysis of Drug Effects

Percent of time subjects spent in the DAC on the Pre-Training Test Day served as the placebo controls, which were compared to those times subjects spent in the DAC during Test Days 1-5, for all pairs of data. With the kind assistance of a fellow faculty member, Dr. Susan Barman, the data were analyzed with a computer-based program designed for complex matrices. This was the Tukey-Kramer Test, GraphPad Instat version 3.05, GraphPad Software, Inc., calculating both within-group and between groups differences of pairs of values (http://www.graphpad.com).

The number of shuttles performed by each rat in each session were also determined from the timer-event recorder tapes. These scores were analyzed for statistical differences using the Student Neumann-Kuels' test. The shuttle responses, then, provided a convenient method of comparing levels of locomotor activity (LMA) for each rat and treatment.

## 3. Results and Discussion

### 3.1. Results

**Figure 2:**
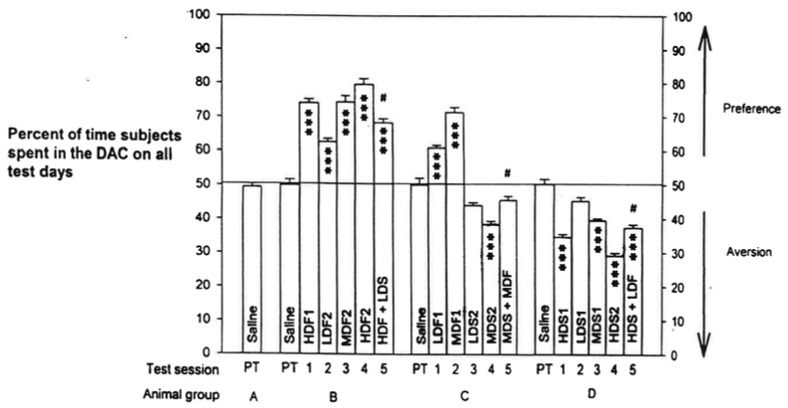
Place-Conditioning Scores (Means +/− SEMs) of Rat Groups Treated with Fentanyl, Spiradoline, and Combinations for the Sequence of the Six Tests Listed in Table 1. Statistical comparisons of group pairs of these scores were done using the Tukey-Kramer Program (see Methods). Scores marked with asterisks are significantly different from saline scores by *p* < *0.*001. Scores without asterisks do not differ from saline scores. The symbol # indicates Test 5 scores differ from Test 4 scores for Groups **B**, **C**, and **D; B** and **D** by *p* < *0.001*, and **C** by *p* < *0.01.* The statistical differences of the remaining critical pairs of scores are presented in Table 2. **Abbreviations:** DAC = Drug-Associated Compartment; PT = Pre-Training Tests with Saline. HDF1 = High-Dose Fentanyl 1, LDS2 = Low-Dose Spiradoline 2, HDF + LDS = combined fentanyl and spiradoline, *etc.*

The place preference of fentanyl and place aversion of spiradoline, and their interactions, yielded a highly significant difference by an overall treatment of all the data, *p* < *0.0001.* The program printout also supplied means +/- standard errors (SEMs) of scores for each group. The Tukey-Kramer Multiple Comparisons Test analyzed all possible pairs of scores for each group to determine within-group and between-groups differences for all Table 1 test scores. These results are graphed in Figure 2.

The analysis indicated significant differences at *p* < *0.001* for 127 pairs of the total of 171 pairs analyzed. Of the remaining 44 pairs, 3 differed by *p* < *0.01*, 11 differed by *p* < *0.05*, and 30 were not significantly different. The numerical means +/− SEMs of scores and the more critical significant differences are listed in Table 2.

Examining within-group comparisons, we established a dose-related preference to fentanyl in Tests 2-4 in Group **B**, though there was no significant difference between High-Dose Fentanyl 1 and Medium-Dose Fentanyl 2. Comparing Group **B**'s High-Dose Fentanyl 2 *vs.* High-Dose Fentanyl + Low-Dose Spiradoline resulted in a difference of *p* < *0.001*. Dose-related preference was also evident to spiradoline in Tests 2-4 for Group **D**, though truncated, since **D**'s Low-Dose Spiradoline 1 was not different than that group's Saline score. High-Dose Spiradoline 2 *vs.* High-Dose Spiradoline + Low-Dose Fentanyl differed by *p* < *0.001*.

In Group **C**, Tests 1-4, there were significant differences for Low-Dose Fentanyl 1 *vs.* Medium-Dose Fentanyl 1 preference and for Low-Dose Spiradoline 2 *vs.* Medium-Dose Spiradoline 2 aversion, both at *p* < *0.001.* Group **C**'s Medium-Dose Spiradoline 2 *vs.* Medium-Dose Fentanyl + Medium-Dose Spiradoline, Test 4 *vs.* Test 5, yielded a significance of *p* < *0.01*. But Test 5 score *vs.* **C**'s Saline score was not significantly different. On pairing Group **C**'s fentanyl scores with that Group's spiradoline scores, the comparisons differed by *p* < *0.001*, as expected.

To reiterate, comparing Test 4 *vs.* Test 5 scores in **B** or **C**, within-group analyses, a high preference by fentanyl in **B** was much reduced when paired with the low dose of spiradoline, and a medium level of aversion by spiradoline in **C** was reversed when paired with the medium dose of fentanyl. Group **C'**s Test 5 score *vs.* Saline did not differ.

Group **D**'s within-group Test 4 *vs.* Test 5 demonstrated that a strong aversive conditioning to spiradoline could be offset by combination with fentanyl, even at a low dose of the MOR agonist. Consider that High-Dose Spiradoline 2 *vs.* Group **D**'s Saline differed by *p* < *0.001*, whereas High-Dose Spiradoline 2 *vs.* High-Dose Spiradoline + Low-Dose Fentanyl differed by only *p* < *0.05.*

Between-groups comparisons, less critical than within-group pairings, still produced results of interest. Pairs of Group **B** *vs.* Group **C** scores differed by *p* < *0.001*, except for non-significance with High-Dose Fentanyl 1 *vs.* Medium-Dose Fentanyl 1, Low-Dose Fentanyl 1 *vs.* Low-Dose Fentanyl 2, Medium-Dose Fentanyl 1 *vs.* Medium-Dose Fentanyl 2, and Medium-Dose Fentanyl 1 *vs.* High-Dose Fentanyl + Low-Dose Spiradoline. All Group **B** *vs.* Group **D** between-groups scores differed by *p* < *0.001*, as anticipated. Fourteen pairs of Group **C** *vs.* Group **D** comparisons differed by *p* < *0.001*, most being fentanyl scores *vs.* spiradoline scores. Two pairs, Low-Dose Spiradoline 2 *vs.* Medium-Dose Spiradoline 1 and Medium-Dose Fentanyl + Medium-Dose Spiradoline *vs.* High-Dose Spiradoline + Low-Dose Fentanyl differed by *p* < *0.05.* The last four pairs, Low-Dose Spiradoline 1 *vs.* Low-Dose Spiradoline 2, Medium-Dose Spiradoline 1 *vs.* Medium-Dose Spiradoline 2, Medium-Dose Spiradoline 2 *vs.* High-Dose Spiradoline + Low-Dose Fentanyl, and Medium-Dose Spiradoline 2 *vs.* Medium-Dose Fentanyl + Medium-Dose Spiradoline, did not differ significantly.

Analyzing pairs of scores that included Saline understandably produced many that were highly significant as well as non-significant. Group **A**, receiving only saline injections throughout the study, registered values not significantly from each other or from Saline scores of the other groups. Of the drug/Saline combinations, only three failed to display significant differences: LDS1 *vs.* any group's Saline score, LDS2 *vs.* any group's Saline score, and MDF + MDS *vs.* any group's Saline score.

**Figure 3:**
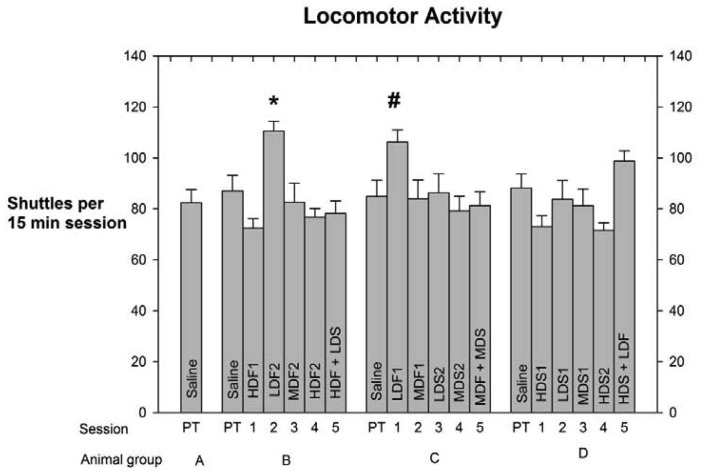
Locomotor Activity (LMA) Scores (Means +/− SEMs) of Rat Groups Treated with Fentanyl, Spiradoline, and Combinations for the Sequence of the Six Tests Listed in Table 1. Statistical comparisons of group pairs of these scores (number of shuttles per group per session) were done by Student Neuman Keuls' Test. The Low-Dose Fentanyl 2 score, Group **B** (*), differed significantly from High-Dose Fentanyl 1, High-Dose Fentanyl 2, High-Dose Spiradoline 1, and High-Dose Fentanyl + Low-Dose Spiradoline by *p* < *0.05*, and from High-Dose Spiradoline 2 by *p* < *0.01*. The Low-Dose Fentanyl 1 score, Group **C** (#), differed significantly from High-Dose Spiradoline 2 by *p* < *0.05*. **Abbreviations:** PT = Pre-Training Tests with Saline.

As indicated above in **Experimental Methods and Materials** the number of shuttles made by each rat in every session was used as an index of the subject's level of locomotor activity (LMA). This measure was calculated to determine any drug-induced effect on LMA that might have compromised the integrity of the PC data. Each group's LMA scores, means +/− SEMs, and levels of significant differences, are depicted in Figure 3.

The analyses of the LMA scores indicate that Low-Dose Fentanyl 2, Group **B**, differed by *p* < *0.05* from the High-Dose Fentanyl 1, High-Dose Fentanyl 2, High-Dose Spiradoline 1, and High-Dose Fentanyl + Low-Dose Spiradoline LMA scores, while the High-Dose Spiradoline 2 LMA score differed by *p* < *0.01*. Also, Low-Dose fentanyl 1 (LDF1) LMA score of Group **C** differed from the HDS2 LMA score, Group **D**, by *p* < *0.05*.

## 3.2. Discussion

In the **Introduction** we reviewed our studies of mu-opioid-receptor (MOR) agonist and kappa-opioid-receptor (KOR) agonist antinociceptions (ANC) in the colorectal distension test (CRD), alone or combined, in cats and rats [7,9,10,12,16]. The purpose for that review was to describe incidental observations of drug-induced behavioral effects that were observed during those experiments. Cats reacted to a MOR agonist alone (oxymorphone) with agitation, excitement, and disorientation. Butorphanol (mixed MOR, KOR agonist [14,15]), given in combination with oxymorphone, calmed the subjects and enhanced the ANC over each single drug effect alone. Cats remained quiet and impassive over the first hour or so after butorphanol injection. During the next hour, however, as the ANC began to wane, a second phase set in, marked by irritability and hypersensitivity to stimuli. This secondary pattern appears to be the emergence of the KOR aversive component of the drug as the MOR component decayed [15].

We also tested butorphanol in dogs [20] for ANC, using the CRD visceral pain model. Behavioral reactions to the drug in dogs were similar to those in cats, excepting mania, while combined MOR, KOR agonists suppressed the KOR-agonist irritability and hypersensitivity.

The place conditioning (PC) apparatus used in this study, with two compartments, is unusual but not unique. It differs from the x-maze used by Briggs in her dissertation research (described above) and from most other multi-compartment PC mazes. Shippenberg *et al.* [2,3] also used a black-white shuttle-box (see below), and Morales *et al.* [21] described a two-compartment PC device as superior to multi-compartment designs for developing place-conditioning responses for opioid drugs. We determined the shuttle-box apparatus to be more efficient for developing PC patterns of MOR and KOR agonists than was the x-maze.

The shuttle-box procedure included an automated technique for recording the subject's PC and locomotor activity (LMA) behaviors. The PC apparatus used by Morales *et al.* [21] also included an automated recording device. Automated recording obviates the need for an observer constantly hovering over the maze, promoting distractions in the subjects as well as subjective errors in judging the subject's responses. Use of two compartments also did not involve neutral transitional areas, where subjects may dwell and dilute the percent of time they spend in designated drug or placebo areas, making PC training less efficient.

A further advantage of the shuttle box format was the ability to measure number of shuttles per session, taken as a measure of LMA. Since LMA responses (Figure 2) were not at all correlated with PC behavior (Figure 1), we were assured that the PC patterns were not confounded by drug-induced LMA effects.

The data in Figure 2 show that some comparisons of drug-pair scores that were not significantly different involved identical drug doses (example: Low-Dose Spiradoline1 *vs.* Low-Dose Spiradoline 2) or those differing by one dose level that appeared to have been skewed by the prior PC training sequence (example: High-Dose Fentanyl 1 *vs.* Medium-Dose Fentanyl 2). The response of Group **C** in test 5 to Medium-Dose Fentanyl + Medium-Dose Spiradoline *vs.* the Test 4 Medium-Dose Spiradoline 2 showed a prominent reduction in the level of spiradoline aversion by adding the medium dose of fentanyl.

The agonists appeared to act reciprocally, or nearly so, in offsetting one-another's opposing motivational effects. This relationship is also supported by Group **B** and Group **D** Test 4 and Test 5 results: a low dose of spiradoline reduced appreciably the preference of a third high dose of fentanyl (**B**) and a low dose of fentanyl suppressed to a large extent the aversion of a third high dose of spiradoline (**C**) (Table 2).

The result that preference occurring with a MOR agonist (fentanyl) was attenuated by a KOR agonist (spiradoline) is certainly not an original observation. This pattern has been reported by a number of investigators [6,22-25]. Several of these authors proposed that this effect may lead to a new therapeutic approach for treatment of MOR-agonist addictions. However, the potential of combining MOR and KOR agonists to suppress the dysphoric effect of KOR agonists, making them more acceptable to patients, has received little recognition. The main obstacle to the clinical application of the KOR-1 agonists spiradoline, enadoline, and U69593 as analgesics has been this prominent dysphoria [2,14,17].

Funada *et al.* [23] reported in mice that U-50,488 (KOR-1 agonist) and E-2078 (dynorphin, endogenous KOR-agonist analogue) developed conditioned place aversion (CPA) in higher doses. Lower (non-aversive) doses still attenuated conditioned place preference (CPP) caused by morphine. These KOR-agonist-induced effects were abolished after pretreatment with nor-binaltorphimine (nor-BNI, KOR-1 antagonist) [26]. Our PC results testing the low dose of spiradoline (non-aversive, see Figure 2) combined with the high dose of fentanyl also showed a prominent decrease in the MOR agonist's CPP. Thus, KOR-agonist suppression of MOR-agonist CPP appears to involve more than a simple cancellation of opposing CPP and CPA states [4].

Shippenberg *et al.* [3], referred to above, subjected naïve rats to hind-limb inflammation, injecting Freund's adjuvant (FA) to produce marked morphine-induced CPP. Rats receiving 7 days of FA exhibited CPP of the same magnitude. Naïve rats receiving U69593 (KOR-1 agonist) manifested CPA. However, rats receiving 7 days of FA failed to develop CPA after U69593 PC training, possibly by activation of an endogenous MOR agonist. If so, their results support our hypothesis (see pg. 3) that opposing rewarding and aversive motivational states interact to cancel each other.

Shippenberg *et al.* also produced CPP with fentanyl and morphine, developing tolerance to the CPP after 4 days of non-contingent morphine [2]. Non-contingent U69593 also caused tolerance to U69593-developed CPA. No cross-tolerance was seen between the MOR- and KOR-agonists. The authors discussed the potential use of KOR agonists as analgesics in chronic pain states, since U69593 CPA was nullified by 7-day FA pretreatment. They suggested that the abolition of this KOR-induced CPA related to a specific alteration in motivational effects of U69593. These results support our proposal that combined MOR and KOR agonists may offer superior pain relief in states of chronic visceral pain [9].

Narita *et al.* [27] noted that patients suffering from cancer pain appeared to develop less than normal psychological dependence upon morphine. They tested for CPP of morphine in control rats and those exposed to inflammation by chronic formalin injections. The inflamed subjects exhibited a suppression of CPP development which was reversed by the KOR-1 antagonist nor-BNI. Their results are further support for our hypothesis that opposing opioid motivational states interact to reciprocally reduce each other.

Narita *et al.* also reduced the dopamine activity in nucleus accumbens by morphine microinjections in control rats when provoking inflammation in the experimental group. This reduction, in turn, was reversed by dopamine A antibody put into nucleus accumbens. They proposed a suppression of morphine reward mechanisms by formalin-induced activation of endogenous KOR agonist systems.

Yamamoto *et al.* [28] found that tolerance development to repeated morphine ANC responses in rats was reversed by U-50,488. Since inhibitory KORs are known to exist in a brain-stem nucleus involving MOR tolerance mechanisms, their activation may have suppressed development of morphine tolerance and maintained ANC.

He and Lee [29] described the restoration of morphine ANC in morphine-tolerant mice by the administration of dynorphin A-(2-17). They suggested that the tolerance depended upon simultaneous activation of MOR sites in the brain and spinal cord, since ANC from intracerebroventricular (i.c.v.) morphine or intrathecal (i.t.) morphine was resistant to tolerance, i.e., MOR/KOR agonist interactions.

Combined i.c.v. and i.t. morphine in naïve animals induced marked ANC synergism. Similar data and concepts were presented by Miatkowski *et al.* [11] in a series of ANC effects of MOR and KOR agonists in rats. The results of both these groups support the hypothesis that systemic KOR-agonist activity reverses systemic MOR-agonist tolerance.

Negus *et al.* [8] performed a recent study analyzing ANC and rewarding/aversive interactions of fentanyl and U69593 in rhesus monkeys, with elements in common with our studies. They described a dose-related ANC for both agonists using warm-water tail-withdrawal (somatic pain model). Combined agonists produced additive ANC, as was the case for the agonists interacting in the cold-water tail-flick in rats reported by our group [7]. It differed from the synergistic ANC observed with the combined drugs using colorectal distension in rats (CRD, a visceral pain model [9]). These differences appear to relate to distinct central nervous system loci sub-serving the somatic *vs.* visceral pain mechanisms (this theme will be expanded below).

The Negus *et al.* article also included interactions of fentanyl and U69593 in schedule-controlled responding for food presentation and in a self-administration fixed-ratio (FR) paradigm. The agonists singly produced rate-decreasing effects in the food presentation, and the combination yielded subadditive effects. Fentanyl, but not U69593, maintained self-administration responding when given singly.

Adding U69593 to fentanyl caused a proportion-dependent decrease in self-administration rates, as well as an increased sensitivity with increasing FR rates (FR strain). It was suggested that the combined agonists reduced abuse liability without compromising the ANC actions. The authors cautioned, however, that the decrease in self-administration rates could have been induced by non-motivational schedule-controlled effects of the drug combination. It was unclear whether or not U69593 aversion was indicated by a reduced self-administration rate below that following placebo (saline) injection.

That very complex PC interactions of MOR and KOR agonists occur at various brain loci is apparent from a study by Sante *et al.* [30]. They microinjected morphine and U-50,488 into the dorsal periaqueductal gray matter of rats tested in a 4-chambered PC procedure. Both agonists induced a CPA. Microinjection of a MOR antagonist (CTOP) did not block these effects, but pretreatment with nor-BNI did. CTOP alone induced CPA, while nor-BNI alone did not.

We observed paradoxical effects of MOR and KOR-agonists and antagonists on ANC in rats tested in CRD [9]. Spiradoline ANC alone was antagonized more by the MOR-specific antagonist beta-funaltrexamine (beta-FNA) than by the KOR-specific antagonist nor-BNI, and the opposite was true for fentanyl. The synergistic ANC of combined agonists was reduced to 50% after beta-FNA and to 25% after nor-BNI.

Fentanyl is a selective MOR agonist and spiradoline is a selective KOR-1 agonist [7]. It is unlikely, then, that any portion of their PC interactions relates to direct effects on the same neural opioid receptors. Neurobiological correlates of opioid-agonist-induced CPP have been postulated to involve dopaminergic and MORs in brain ventral striatum and other mesolimbic areas associated with development of preference. Aversive responses are thought to entail dynorphin (endogenous kappa opioid) receptors and cholinergic receptors in the extended amygdala and other mesocortical sites [31,32].

Evidence that the motivational interactions of MOR and KOR agonists as described above may apply to humans was furnished by Preston and Bigelow [33]. They induced MOR agonist activity (hydromorphone) and KOR plus MOR activity (pentazocine) in human volunteers. A 25 mg dose of naltrexone (non-selective opioid antagonist) blocked both MOR- and KOR-agonist effects. However, a 12.5 mg dose of naltrexone decreased the MOR-agonist effect of pentazocine and uncovered greater KOR-agonist activity of pentazocine (dysphoria, psychotomimetic influences). Thus, simultaneous MOR and KOR agonist effects can result in suppression of KOR-agonist aversive side effects in man, in agreement with the results of the present study in rats and in other studies [3, 4].

## 4. Conclusions

The above-presented data suggest several types of MOR- and KOR-agonist interactions:

(1) Dose-related place preference to fentanyl and dose-related place aversion to spiradoline were observed in the shuttle-box test model. These same dose ranges of the agonists were previously shown to induce dose-related additive [7] and synergistic [9] antinociception in Cold-Water Tail-Flick and colorectal distension, respectively.
(2) Combined agonists appeared to result in a mutual antagonism of MOR-agonist preference and KOR-agonist aversion, offsetting one-another. Proposed neural mechanisms for these opposing motivational response patterns include neuronal components in brainstem nuclei, mesolimbic and mesocortical circuits, and frontal-brain extended amygdalar complex, competing to express euphoric or dysphoric states ofmind [3,4,5,22,23,27,29,30,31,32,33].
(3) The neural substrate for colorectal distension antinociceptive interactions of the agonists appears to differ from the above, involving neural spinal/supraspinal chains containing interspersed MOR and KOR units to support a synergistic antinociception [34,35,37,38,39].
(4) The reciprocal suppressions of MOR-agonist preference and KOR agonist aversion with combined agonists suggests a clinical potential for improved control of pain in human patients, and, in particular, chronic visceral pain syndromes. Combining the agents in this manner should reduce the levels of adverse side effects, including KOR agonist dysphoria, and the risk of MOR-agonist addiction, tolerance, dependence, and hyperalgesia.

In the latter part of the last century the conventional wisdom concerning attempts to combine opioid drugs for greater and safer pain relief was that the concept had no merit [40,41,42]. This conclusion was apparently based mainly upon results of experimental somatic pain studies and agonist-antagonist characteristics of the earlier kappa-opioid agonist's profiles (pentazocine, nalbuphine). We propose that the data presented above indicates that this judgment was premature.

## Figures and Tables

**Table 1:** Schedule of training and test days, drugs and placebo sessions, for all four groups.

**Sessions**	**Days**	**Group A**	**Group B**	**Group C**	**Group D**
**Pre-training**	1, 2, 4, 6 in DAC (a)	S (c)	S	S	S
	3,5 in PAC (b)	S	S	S	S
**Pre-training test**	7	S	S	S	S
**Training**	8, 9, 11, 13 in DAC	S	High-Dose	Low-Dose	High-Dose
**Session 1**			Fentanyl 1	Fentanyl 1	Spiradoline 1
	10, 12 in PAC	S	S	S	S
**Test Day1**	14	S	S	S	S
**Training**	15, 16, 18, 20 in DAC	S	Low-Dose	Medium-Dose	Low-Dose
**Session 2**			Fentanyl 2	Fentanyl 1	Spiradoline 1
	17, 19 in PAC	S	S	S	S
**Test Day 2**	21	S	S	S	S
**Training**	22, 23, 25, 27 in DAC	S	Medium-Dose	Low-Dose	Medium-Dose
**Session 3**			Fentanyl 2	Spiradoline 2	Spiradoline 1
	24, 26 in PAC	S	S	S	S
**Test Day 3**	28	S	S	S	S
**Training**	29, 30, 32, 34 in DAC	S	High-Dose	Medium-Dose	High-Dose
**Session 4**			Fentanyl 2	Spiradoline 2	Spiradoline 1
	31, 33 in PAC	S	S	S	S
**Test Day 4**	35	S	S	S	S
**Training**	35, 37, 39, 41 in DAC	S	HDF + LDS	MDF + MDS	HDS + LDSF
**Session 5**			(d)	(d)	(d)
	38, 40 in PAC	S	S	S	S
**Test Day 5**	42	S	S	S	S

(a) = Subjects restricted to drug-associated compartment; (b) = Subjects restricted to placebo-associated compartment; (c) = Saline; (d) = High-dose fentanyl plus Low-dose spiradoline; Medium-dose fentanyl plus Medium-dose spiradoline; High-dose spiradoline plus Low-dose fentanyl.

**Table 2:** Place-Conditioning Scores: I. Means +/− SEMs derived from comparing group Saline values with Group Fentanyl, Spiradoline, or combination values for Sequences of Saline and drug training as listed in Table1, using the Tukey-Kramer program of statistical analysis. II. Statistical comparisons of drug/drug pairs for both Within-Group comparisons or Between-Groups comparisons of most critical pairs.

I. **Group**	**Group A**	**Group B**	**Group C**	**Group D**
**Pre-training**	Saline	Saline	Saline	Saline
**Day 7:**	49.53+/−1.00 (a)	49.96+/−1.64	50.05+/−1.99	50.27+/−1.45
**Test 1**	Saline	High-Dose	Low-Dose	High-Dose
Day 14:		Fentanyl 2	Fentanyl 1	Spiradoline 1
		74.07+/−1.17	60.93+/-0.72	34.67+/−0.87
**Test 2**	Saline	Low-Dose	Medium-Dose	Low-Dose
Day 21:		Fentanyl 2	Fentanyl 1	Spiradoline 1
		62.63+/−0.98	71.38+/−1.49	45.28+/−1.15
**Test 3**	Saline	Medium-Dose	Low-Dose	Medium-Dose
Day 28:		Fentanyl 2	Spiradoline 2	Spiradoline 1
		74.32+/−1.91	44.08+/−0.77	39.58+/−0.60
**Test 4**	Saline	High-Dose	Medium-Dose	High-Dose
Day 35:		Fentanyl 2	Spiradoline 2	Spiradoline 2
		79.67+/−1.69	38.37+/−0.97	29.10+/−0.87
**Test 5**	Saline	High-Dose	Medium-Dose	High-Dose
Day 42:		Fentanyl plus	Fentanyl plus	Spiradoline
		Low-Dose	Medium-Dose	plus Low-
		Spiradoline	Spiradoline	Dose Fentanyl
		68.28+/−1.23	45.56+/−1.18	37.38+/−1.01

II. Within-Group comparisons of drug/drug pairs of scores: Group B pairs differ by *p* < 0.001, except for HDF1 *vs.* MDF2 (n.s.). Group C pairs differ by *p* < 0.001, excepting MDS2 *vs.* MDF + MDF (*p* < 0.05), and LDS2 *vs.* MDF + MDS (n.s.). Group D differ by *p* < 0.001, excepting HDS2 *vs.* MDS1 and HDS1 *vs.* HDS2 (*p* < 0.01), LDS1 *vs.* MDS1 and HDS2 *vs.* HDS + LDF (*p* < 0.05), and HDS1 *vs.* HDF + LDF and MDS1 *vs.* HDS + LDF (n.s.).

Between-Group comparisons of drug/drug pairs of scores: Group B *vs.* Group C pairs differ by *p* < 0.001, excepting HDF2 *vs.* MDF1 (*p* < 0.05), and HDF1 *vs.* MDF1, LDF2, and MDF2 *vs.* MDF1 (n.s.). Group B *vs.* Group D pairs differ by *p* < 0.001. Group C *vs.* Group D pairs differ by *p* < 0.001, except for LDS2 *vs.* MDS1, LDS1 *vs.* MDS2, MDS2 *vs.* HDS1, and MDF + MDS *vs.* HDS + LDF (*p* < 0.05), and LDS1 vs, LDS2, MDS2 *vs.* HDS + LDF, MDS1 *vs.* MDS2, and LDS1 *vs.* MDF + MDS (n.s.).

Saline pairs were all non-significant (n.s.). Within-group comparisons derive from vertical columns, between-group comparisons from horizontal or lateral columns of I. Abbreviation codes for II: LDF = Low-Dose Fentanyl; MDF = Medium-Dose Fentanyl; HDF = High-Dose Fentanyl; LDS = Low-Dose Spiradoline; MDS = Medium-Dose Spiradoline; HDS = High-Dose Spiradoline; *etc.*
